# EEG signatures of mindfulness-based artistic–contemplative sound interventions: a pilot study on aesthetic states of consciousness

**DOI:** 10.3389/fpsyg.2026.1846392

**Published:** 2026-07-28

**Authors:** Hercules Morais, Débora Regina de Aguiar, Matheus Hissa Lourenço Ferreira, Patrícia Zocchi dos Santos, Juliana Zellauy, Ana Cristina Fraga da Silva Schwingel, Paulina Lamas-Morales, Javier García Campayo, Yolanda López del Hoyo, Marcelo Demarzo

**Affiliations:** 1 Department of Preventive Medicine, Escola Paulista de Medicina, Federal University of São Paulo (UNIFESP), São Paulo, Brazil; 2 Area of Basic Psychology, Faculty of Social and Labour Sciences, University of Zaragoza, Zaragoza, Spain; 3 Program in Evidence-Based Health, Department of Medicine, Federal University of São Paulo (UNIFESP), São Paulo, Brazil; 4 EEFFTO, Sport Sciences, Federal University of Minas Gerais (UFMG), Belo Horizonte, Brazil; 5 Faculty of Medicine, Hospital das Clínicas, University of São Paulo (HCFMUSP), São Paulo, Brazil; 6 Escola de Ciências da Saúde e da Vida, Departamento de Neurociências e Comportamento, Pontifical Catholic University of Rio Grande do Sul (PUCRS), Porto Alegre, Brazil; 7 Training and Development Center of the Chamber of Deputies (CEFOR), Brasília, Brazil; 8 Department of Medicine, Psychiatry and Dermatology, Faculty of Medicine, University of Zaragoza, Zaragoza, Spain; 9 Mente Aberta - The Brazilian Center for Mindfulness and Health Promotion, Escola Paulista de Medicina, Federal University of São Paulo (UNIFESP), São Paulo, Brazil; 10 Consórcio Acadêmico Brasileiro de Saúde Integrativa (CABSIN), São Paulo, Brazil

**Keywords:** aesthetic experience, artistic interventions, contemplative neuroscience, electroencephalography, mindfulness, neuroaesthetics

## Abstract

**Background:**

Mindfulness-based artistic–contemplative interventions, including auditory “sound pills,” have increasingly been investigated using neurophysiological measures such as electroencephalography (EEG) to promote psychological well-being and attentional regulation. However, the neurophysiological mechanisms underlying these interventions remain poorly understood.

**Methods:**

This pilot, exploratory, within-subject proof-of-concept study investigated the neurophysiological effects of mindfulness-based artistic–contemplative sound pills in adults using EEG. Twelve participants were exposed to structured auditory interventions integrating poetic narratives, soundscapes, and mindfulness instructions. EEG activity was recorded before, during, and after the intervention, and spectral power across frequency bands was analyzed using linear mixed-effects models. Subgroup analyses by artistic modality (visual arts, dance, music, and theater) were also performed.

**Results:**

At the group level, exploratory analyses indicated a statistically significant modulation of high-frequency beta activity (20–30 Hz), characterized by a reduction during the intervention compared to the pre-intervention baseline, followed by a return toward baseline levels in the post-intervention phase. No statistically significant global changes were observed in the other frequency bands examined, including alpha (8–12 Hz), theta (4–8 Hz), delta (0.5–4 Hz), mu, low-frequency beta (12–20 Hz), and the combined theta + alpha band. Subgroup analyses by artistic modality suggested descriptive, modality-specific oscillatory patterns; however, these findings should be interpreted as hypothesis-generating only, given the very small sample sizes per modality (n = 2–4) and the absence of a control condition.

**Conclusion:**

Overall, the results provide preliminary evidence that artistic–contemplative auditory interventions can modulate neural oscillatory dynamics, particularly within the high-frequency beta range. Given the small sample size, within-subject design, and lack of a control condition, these findings should be interpreted with caution and require confirmation in larger controlled studies.

## 1 Introduction

Although mindfulness-based interventions and arts-based practices have independently shown benefits for psychological well-being and attentional regulation, their combined effects on brain activity remain poorly understood. To date, few electroencephalography (EEG) studies have examined whether brief, standardized auditory interventions that integrate poetic narrative, immersive soundscapes, and mindfulness instructions can modulate neural oscillatory dynamics in adults. This gap is relevant because such interventions may offer an accessible and scalable way to induce states of internally directed attention and aesthetic engagement, yet their neurophysiological mechanisms have not been systematically investigated. Addressing this gap is essential for advancing the integration between contemplative neuroscience and neuroaesthetics and for informing the development of novel mental health interventions.

In a contemporary context marked by psychological exhaustion, attentional fragmentation, and a crisis of meaning among young adults, the development of interventions that integrate science, art, and contemplation has become increasingly urgent (Sonke et al., 2009; World Health Organization, 2022). Rapid digital transformation and the demands of hyperconnected daily life have contributed to rising levels of stress, anxiety, and difficulties in emotional self-regulation, particularly among young populations (Twenge et al., 2018; Orben and Przybylski, 2019). In this context, mindfulness-based practices have emerged as evidence-based approaches for promoting psychological well-being, enhancing attentional regulation, and reducing stress-related symptoms (Kabat-Zinn, 2003; Creswell, 2017). However, despite the growth of these interventions, studies investigating their effects when integrated with structured artistic stimuli and assessed through direct neurophysiological measures, such as EEG, remain limited (Lomas et al., 2015).

Existing evidence-based mindfulness interventions, such as MBSR and MBCT, have demonstrated neurophysiological effects including increased alpha and theta power (Tang et al., 2015). Arts-based health interventions have shown benefits for mood, stress, and social connectedness (Clift, 2020), but rarely involve neurophysiological assessment. Neither tradition has systematically exploited the convergence of symbolic narrative, soundscape design, and contemplative instruction in a single, brief, standardized, and potentially scalable auditory format. The key potential advantages of sound pills over standard mindfulness programs are their brevity (approximately 10 min), accessibility (audio format requiring no in-person delivery), and integration of artistic engagement as a mechanism for deepening contemplative absorption.

The intersection between mindfulness practices and artistic experiences remains a relatively underexplored research area, particularly when simultaneous neurophysiological measurements are considered (Brattico and Pearce, 2013; Chatterjee and Vartanian, 2014). Interventions integrating symbolic language, immersive soundscapes, and attention-guiding instructions may facilitate the emergence of aesthetic states of consciousness, promoting perceptual immersion, reflective self-awareness, and processes of meaning construction (Brattico and Pearce, 2013; Chatterjee and Vartanian, 2014). When delivered through accessible formats such as audio-guided interventions, these practices may have promising applications in integrative mental health, educational contexts, and broader human development settings (Creswell, 2017; Clift, 2020).

Electroencephalography (EEG) is a valuable method for investigating mental states induced by contemplative and aesthetic practices due to its high temporal resolution and sensitivity to neural oscillations associated with cognitive and affective processes (Cahn and Polich, 2006; Lomas et al., 2015). Previous studies associate the theta band (4–8 Hz) with internally directed attention, memory processes, and imaginative cognition (Klimesch, 1999). The alpha band (8–12 Hz), in turn, has been associated with relaxed wakefulness, functional inhibition of sensory processing, and internally oriented attentional states (Jensen and Mazaheri, 2010; Klimesch, 2012). The presence of delta activity (0.5–4 Hz) during wakefulness has been associated with internally oriented processing as well as emotional and motivational dynamics (Knyazev, 2007; Harmony, 2013). Higher-frequency oscillations, particularly in the beta (12–30 Hz) and gamma (30–100 Hz) ranges, have been associated with cognitive engagement, attentional processes, and large-scale neural information integration (Fries, 2009; Engel and Fries, 2010). Thus, in interventions aimed at inducing contemplative or aesthetic states, a homogeneous modulation across all frequency bands is not necessarily expected, as distinct oscillatory frequencies reflect different and often complementary neurocognitive functions (Başar et al., 2001; Buzsáki and Draguhn, 2004). Within the beta range, frequency subdivisions have been associated with distinct aspects of cognitive control, self-regulation, and attentional monitoring, suggesting that more fine-grained analyses of this band may provide relevant insights into transitions between externally oriented engagement and internally sustained attention (Engel and Fries, 2010; Spitzer and Haegens, 2017).

Based on the contemplative neuroscience literature, the *a priori* primary targets of this study were the theta (4–8 Hz) and alpha (8–12 Hz) bands. Theta oscillations have been consistently associated with internally directed attention, working memory, and imaginative cognition (Klimesch, 1999), processes that the structured narrative and symbolic content of the sound pills were specifically designed to engage. Alpha power, particularly in posterior regions, has been associated with relaxed wakefulness and functional inhibition of externally oriented sensory processing (Jensen and Mazaheri, 2010), states compatible with the contemplative and receptive stance encouraged by the intervention. Higher-frequency oscillations (beta and gamma) were examined as secondary outcomes, given their roles in cognitive engagement and large-scale information integration (Fries, 2009; Engel and Fries, 2010).

Concurrently, the field of neuroaesthetics has increasingly investigated the neural mechanisms underlying artistic experiences, suggesting that they involve cognitive–affective processes related to aesthetic absorption, immersive engagement, and meaning-making (Vessel et al., 2012; Leder and Nadal, 2014; Pearce et al., 2016). These findings contribute to understanding aesthetic experience as a dynamic and multifaceted phenomenon; however, the literature still lacks investigations that systematically integrate structured artistic interventions, subjective experiential states, and objective neurophysiological measures (Chatterjee and Vartanian, 2014; Pelowski et al., 2017).

Despite these advances, a significant gap remains in the literature regarding the neurophysiological effects of structured artistic–contemplative interventions designed as “sound pills”—auditory compositions integrating poetic narrative, musical soundscapes, and mindfulness-based instructions. Such formats remain scarcely examined from an EEG perspective but may offer a standardized and reproducible means of inducing aesthetic states of consciousness by combining narrative engagement, auditory stimulation, and contemplative guidance (Brattico and Pearce, 2013; Koelsch, 2014). Investigating these effects in adults is particularly relevant given the growing prevalence of psychological distress in this population and the increasing demand for accessible, evidence-based mental health interventions (Auerbach et al., 2016; World Health Organization, 2022).

Existing mindfulness-based programs such as MBSR and MBCT have demonstrated neurophysiological effects on EEG, including increases in alpha and theta power (Lomas et al., 2015; Tang et al., 2015). Arts-based interventions have shown benefits for psychological well-being (Clift, 2020), yet most require multiple in-person sessions and substantial time commitment, limiting their accessibility. Furthermore, few interventions systematically integrate structured artistic stimuli with contemplative guidance within a brief, standardized auditory format. The present study addresses this gap by evaluating short (~10-min), pre-recorded artistic–contemplative “sound pills” that combine symbolic narrative, soundscape, and mindfulness instructions.

For the purposes of this study, we define aesthetic states of consciousness as transient neurocognitive states that may emerge during engagement with immersive artistic–contemplative stimuli. These states are characterized by a shift toward internally directed attention, reduced processing of external sensory information, and increased engagement with symbolic, emotional, and self-referential content. We operationalize these states through changes in EEG spectral power, with particular interest in increases in theta (4–8 Hz) and alpha (8–12 Hz) activity, although modulation in other frequency bands may also occur depending on the nature of the stimulus. This construct is related to, but distinct from, flow states (Csikszentmihalyi, 2009) and default mode network activation during aesthetic experiences (Vessel et al., 2012). It is used here as a provisional heuristic framework whose boundaries and empirical validity require further investigation.

In light of this gap, we hypothesized that exposure to mindfulness-based artistic–contemplative sound pills would modulate neural oscillatory activity, with distinct patterns across frequency bands reflecting their functional roles. While increases in theta and alpha activity were hypothesized based on the contemplative neuroscience literature, the integrative and symbolically structured nature of the stimulus may also be associated with modulation in higher-frequency bands related to cognitive engagement and large-scale information integration (Cahn and Polich, 2006; Fries, 2009; Engel and Fries, 2010; Lomas et al., 2015).

The aim of this study was to investigate the neurophysiological effects of listening to mindfulness-based artistic–contemplative sound pills on neural oscillatory activity measured by EEG in adults.

## 2 Materials and methods

### 2.1 Ethical considerations

This study was approved by the Research Ethics Committee/Plataforma Brasil - UNIFESP under CAAE 40149120.4.0000.5505 on March 4, 2021. The study was registered retrospectively in the Brazilian Registry of Clinical Trials (ReBEC) under the number RBR-68qv6wf on June 25, 2025. Data collection was conducted following ethics approval, between February 20 and 23, 2025. The retrospective registration is acknowledged as a limitation of the study’s registration rigor.

### 2.2 Study design

Exploratory study with a quasi-experimental within-subject design and a quantitative approach. EEG, a noninvasive neurophysiological technique that records brain electrical activity generated by neuronal populations, was employed in this study to investigate the neurophysiological effects of mindfulness-based artistic–contemplative practices on the electroencephalographic activity of adults. Recordings were conducted before, during, and after listening to mindfulness-based artistic–contemplative sound interventions. A detailed description of the equipment and its technical specifications is presented below.

### 2.3 Participants

A total of 12 participants aged between 18 and 66 years were included in the study. All participants provided written informed consent prior to participation. The study followed the ethical principles established by CNS Resolution No. 466/2012. The broad age range was considered acceptable in this exploratory pilot study because the primary objective was to evaluate protocol feasibility in a heterogeneous adult convenience sample. Nevertheless, well-documented age-related changes in EEG activity, including alterations in alpha frequency, spectral power distribution, and other electrophysiological characteristics across the lifespan (Dustman et al., 1993; Klimesch, 1999), may have introduced variability that limits neurophysiological comparability between participants (see Limitations).

### 2.4 Instruments

#### 2.4.1 Interventions—artistic–contemplative sound pills

Each participant was exposed to a single sound pill belonging to one of five artistic modalities. The modalities were not administered together or in combination; each participant received only one sound pill in a single session. Thus, the five modalities represent five distinct experimental conditions within the study, allocated across participants according to a predefined sequence criterion. The sound pills were developed by the author-researcher based on original texts, narrated in audio format in Brazilian Portuguese, and edited with soundtrack and sound effects. Each piece (~10 min) belonged to one of five modalities: theater, dance, music, literature, and visual arts. The content combines subtle mindfulness instructions, symbolic language, and contemplative soundscapes, functioning as a standardized auditory stimulus for inducing aesthetic states of consciousness. Audio specifications, including file format (WAV/MP3), sampling rate, bitrate, loudness (LUFS), and headphones with standardized volume, were standardized across all recordings.

Each modality was designed with specific neuroaesthetic and existential expectations. The visual arts piece was intended to evoke visual imagery and symbolic processing, potentially engaging secondary visual networks and affective circuits associated with meaning-making and cognitive flexibility. The dance-based intervention emphasized interoceptive and sensorimotor imagery without physical movement, aiming to strengthen body–awareness integration, proprioception, and embodied self-regulation. The literary modality sought to activate semantic, autobiographical memory, and constructive imagination networks, fostering reflective processing of complex emotions and narrative integration of the self. The music-based piece explored rhythm and temporality as metaphors for lived experience, potentially engaging auditory, emotional, and self-referential networks involved in affective resonance and temporal awareness. Finally, the theatrical modality incorporated dramatic language and metacognitive prompts to stimulate perspective-taking, mentalization, and reflective awareness.

Synopses, full scripts, and technical editing protocols are available in the Supplementary material.

### 2.5 Electroencephalographic recording (EEG)

Electroencephalographic recording was performed using a 19-channel system, following the international 10–20 system for electrode placement. Electrode impedances were maintained below 10 kΩ. During acquisition, participants remained seated in a quiet environment with controlled lighting. The reference used was the combination of electrodes A1 and A2 (linked mastoids), and the ground electrode was positioned at CP6. The signal sampling rate was 1,000 Hz. For signal processing, the following filters were applied: a high-pass filter (HPF) at 0.05 Hz, a low-pass filter (LPF) at 70 Hz, and a 60 Hz notch filter to remove electrical line interference. The equipment used for acquisition was the Neuron-Spectrum 3 amplifier (Kandel), operating with the Neurosoft system. An initial verification was conducted to inspect noise and perform adjustments prior to the start of recordings.

### 2.6 Procedures and temporal sequence

Upon arrival, participants received standardized explanations about the study, followed by a brief anamnesis and the signing of the informed consent form. Subsequently, sociodemographic data were collected. Throughout the entire procedure, participants were seated in a comfortable chair in a quiet room with controlled lighting.

Temporal sequence of the session—Eyes open (baseline 1): 5 min; Eyes closed (baseline 2): 5 min; During the sound pill (eyes closed): 10 min of continuous EEG; Post (eyes open): 5 min; Post (eyes closed): 5 min. Transitions were signaled by brief verbal commands.

### 2.7 Preprocessing and spectral analysis

After the preparation procedure, the EEG signal was monitored until it was considered stable for normative recording. For artifact removal, segments containing noise such as blinks, eye movements, and other artifacts were identified using an amplitude threshold of ±100 microvolts (μV) and excluded. This procedure was complemented by visual inspection to ensure the removal of residual artifacts, including muscle activity and movement-related noise.

The raw data were segmented by experimental condition (Eyes Open, Eyes Closed, During the Sound Pill, and Post-Intervention), with each condition block lasting 5–10 min. For spectral estimation, the continuous data were divided into non-overlapping 2-s epochs. Spectral estimation was performed using the Fast Fourier Transform (FFT). It is important to note that high-frequency beta (20–30 Hz) is the frequency range most susceptible to EMG contamination from scalp and neck muscles (Goncharova et al., 2003; Shackman et al., 2009). While amplitude-based artifact rejection was applied, residual low-amplitude EMG activity cannot be fully excluded, and this represents an unresolved potential confound for the beta HF findings. Future studies should employ independent component analysis (ICA) for more rigorous EMG separation.

The primary metric used for analysis was relative power by frequency band: delta (0.5–4 Hz), theta (4–8 Hz), alpha (8–12 Hz), beta (12–30 Hz), and gamma (30–100 Hz). For exploratory analyses, the beta band was subdivided into low-frequency beta (beta LF: 12–20 Hz) and high-frequency beta (beta HF: 20–30 Hz). Regions of interest: Frontal (Fp1, Fp2, F7, F3, Fz, F4, F8), Central (C3, Cz, C4), Temporal (T3, T4, T5, T6), Parietal (P3, Pz, P4), Occipital (O1, O2).

### 2.8 Statistical analysis and data structure

All statistical analyses were conducted in accordance with current recommendations for exploratory electroencephalography (EEG) studies employing a repeated-measures design and a small sample size. The analyses were predominantly hypothesis-driven for the theta (4–8 Hz) and alpha (8–12 Hz) bands, whereas the delta band (0.5–4 Hz) and higher-frequency bands were examined secondarily and exploratorily.

To facilitate statistical analysis and align with the primary study objectives, the five experimental recording phases were consolidated into three analytical conditions: “before,” “during,” and “after.” The “before” condition combined the eyes-open and eyes-closed baseline recordings; the “after” condition combined the post-intervention eyes-open and eyes-closed recordings.

The primary results, based on these aggregated conditions, are presented in Supplementary Table S1. To evaluate the robustness of the findings with respect to the aggregation of eyes-open and eyes-closed recordings, we additionally conducted disaggregated analyses comparing the original five conditions separately. These contrasts are reported in Supplementary Table S2 as a robustness check. The eyes-closed baseline versus during contrast is considered the theoretically most appropriate comparison for the alpha-band primary outcome.

### 2.9 Main inferential models

Analyses were conducted using the three predefined conditions. Statistical inference was based on Linear Mixed-Effects Models (LMM). Experimental condition and region were treated as fixed effects, while participant was included as a random effect (random intercept). Separate models were fitted for each frequency band.

### 2.10 Planned contrasts

*A priori* contrasts were specified to address the primary study hypotheses, focusing on: (1) differences between the intervention period and the eyes-closed baseline condition, and (2) differences between the intervention period and the post-intervention period.

### 2.11 Effect size and control of multiple comparisons

The primary outcomes were relative power in the theta (4–8 Hz) and alpha (8–12 Hz) frequency bands. Secondary outcomes included delta power and derived metrics (e.g., combined theta + alpha and beta sub-bands). Effect sizes were reported alongside *p* values. For mixed models, Cohen’s dz (standardized mean difference for within-subject paired comparisons) was calculated and is reported throughout using a uniform sign convention: positive values indicate higher values in the first condition named in the contrast (e.g., ‘Before’ in ‘Before – During’), whereas negative values indicate higher values in the second condition. This convention follows directly from the labeling of the pairwise comparisons (first condition minus second condition). All analyses correspond to paired contrasts derived from the linear mixed-effects model.

For the primary analyses, the false discovery rate (FDR) procedure was applied within each frequency band to control for multiple comparisons, and the results are presented in Supplementary Table S1. In the disaggregated robustness analyses (Supplementary Table S2), uncorrected nominal *p* values are reported to facilitate transparent evaluation of individual contrasts. Statistical significance for the primary outcomes was determined based on FDR-corrected results from the aggregated models.

### 2.12 Subgroup and exploratory analyses

Additional analyses were conducted to explore potential differences in oscillatory patterns across the five artistic modalities (music, dance, literature, theater, and visual arts). These analyses were considered exploratory, and hypothesis-generating given the small and uneven sample sizes across modalities. Emphasis was placed on the descriptive characterization and directional consistency of observed changes across experimental conditions rather than on formal statistical inference. The literature modality was represented by a single participant and was not subjected to subgroup analysis; consequently, no inferential interpretation was attempted for this modality.

### 2.13 Software and reproducibility

All analyses were performed in R (version 4.5.1) (R Core Team, 2025) within the RStudio environment (Posit Team, 2024). Linear mixed-effects models were fitted using appropriate statistical packages (lme4, emmeans). Given the exploratory and pilot nature of the study, no *a priori* power calculation was conducted.

## 3 Results

### 3.1 Demographic and academic profile

A total of 12 participants were included in the analysis. The mean age was 25.2 ± 13.0 years (range: 18–66 years). The sample exhibited a skewed distribution regarding sex, with males representing the majority (67%, *n* = 8) compared to females (33%, *n* = 4). A high proportion of participants were single (92%, *n* = 11). Racially, the sample was predominantly white (75%, *n* = 9). Regarding professional status, the majority were students (83%, *n* = 10). Participants were categorized according to the sound pill modality as Music (33%, *n* = 4), Dance (25%, *n* = 3), Theater (17%, *n* = 2), Visual Arts (17%, *n* = 2), and Literature (8%, *n* = 1) (Table 1).

**Table 1:** Sociodemographic and clinical characteristics of participants by sound pill modality (*N* = 12).

Variable	Dance (*n* = 3)	Literature (*n* = 1)	Music (*n* = 4)	Theater (*n* = 2)	Visual arts (*n* = 2)	*p*-value
Age (years)	23 (3)	20 (NA)	21 (1)	45 (29.7)	20 (3)	0.20
Sex						>0.90
Female	1 (33%)	0 (0%)	2 (50%)	1 (50%)	0 (0%)	
Male	2 (67%)	1 (100%)	2 (50%)	1 (50%)	2 (100%)	
Marital status						0.40
Married	0 (0%)	0 (0%)	0 (0%)	1 (50%)	0 (0%)	
Single	3 (100%)	1 (100%)	4 (100%)	1 (50%)	2 (100%)	
Race/ethnicity						0.60
Brown	1 (33%)	1 (100%)	1 (25%)	0 (0%)	0 (0%)	
White	2 (67%)	0 (0%)	3 (75%)	2 (100%)	2 (100%)	
Household size	1.33 (0.58)	6.00 (NA)	2.75 (1.71)	3.00 (1.41)	2.00 (1.41)	0.30
Graduation major						0.020
Business/admin	2 (67%)	0 (0%)	4 (100%)	1 (50%)	0 (0%)	
Economics	0 (0%)	1 (100%)	0 (0%)	0 (0%)	2 (100%)	
Other/no answer	1 (33%)	0 (0%)	0 (0%)	0 (0%)	0 (0%)	
Pedagogy	0 (0%)	0 (0%)	0 (0%)	1 (50%)	0 (0%)	
Profession						0.70
Intern	0 (0%)	0 (0%)	1 (25%)	0 (0%)	0 (0%)	
Student	3 (100%)	1 (100%)	3 (75%)	1 (50%)	2 (100%)	
Teacher	0 (0%)	0 (0%)	0 (0%)	1 (50%)	0 (0%)	
Employment status						>0.90
Unemployed	2 (67%)	1 (100%)	3 (75%)	1 (50%)	1 (50%)	
Working	1 (33%)	0 (0%)	1 (25%)	1 (50%)	0 (0%)	
No answer	0 (0%)	0 (0%)	0 (0%)	0 (0%)	1 (50%)	
Mental health diagnoses						
Depression	1 (33%)	0 (0%)	0 (0%)	0 (0%)	1 (50%)	0.60
Anxiety	3 (100%)	0 (0%)	1 (25%)	0 (0%)	2 (100%)	0.061
Stress	1 (33%)	0 (0%)	0 (0%)	0 (0%)	1 (50%)	0.60
ADHD	0 (0%)	0 (0%)	1 (25%)	0 (0%)	1 (50%)	0.80
Current treatment						0.30
Yes	3 (100%)	0 (0%)	2 (50%)	0 (0%)	1 (50%)	
No	0 (0%)	1 (100%)	1 (25%)	2 (100%)	1 (50%)	
No answer	0 (0%)	0 (0%)	1 (25%)	0 (0%)	0 (0%)	
Use of controlled medication	3 (100%)	0 (0%)	2 (50%)	2 (100%)	1 (50%)	0.40

Values are presented as Mean (SD) or *n* (%). ADHD, Attention-Deficit/Hyperactivity Disorder. Between-group comparisons should be interpreted cautiously due to the small subgroup sizes and exploratory nature of the analysis.

### 3.2 Mental health diagnostics

Anxiety was the most frequently reported diagnosis, present in 50% (*n* = 6) of the cohort. Depression, Stress, and ADHD were each reported by 17% (*n* = 2) of the participants. 50% (*n* = 6) reported being in current treatment, and 67% (*n* = 8) reported the use of controlled medication. The high prevalence of controlled medication use (67%) is acknowledged as a potential confound for EEG spectral power findings, particularly for the beta and alpha bands, given the well-documented effects of psychotropic medications on EEG activity (Blume, 2006). This limitation is discussed in detail in Section 4.

### 3.3 Comparative profile

No significant differences were detected across groups for sex (*p* > 0.90), marital status (*p* = 0.40), or race/ethnicity (*p* = 0.60). A statistically significant difference was found for graduation major (*p* = 0.020). A borderline difference in anxiety prevalence was observed across modalities (*p* = 0.061), with 100% anxiety prevalence in the dance and visual arts subgroups. These findings raise the possibility that differences in clinical profiles across modalities, particularly in anxiety prevalence, may have contributed to the observed oscillatory patterns. This interpretation is considered in the Discussion section.

### 3.4 Overall analysis

The primary outcomes were relative power in the theta and alpha frequency bands. After FDR correction, no statistically significant differences were observed between experimental conditions for either outcome, and all effect sizes were small or trivial.

For the alpha rhythm, no significant differences were observed between the before and during conditions (mean difference = −0.27; 95% CI −5.94 to 5.39; *p* = 0.921; dz = −0.02) or between the during and after conditions (mean difference = 1.94; 95% CI −3.73 to 7.60; *p* = 0.486; dz = +0.15) (Supplementary Table S1).

For the theta rhythm, no statistically significant differences were observed after FDR correction. Although the before versus after comparison reached nominal significance (*p* = 0.044), this effect did not remain significant following correction for multiple comparisons (FDR-adjusted *p* = 0.133).

**Figure 1:**
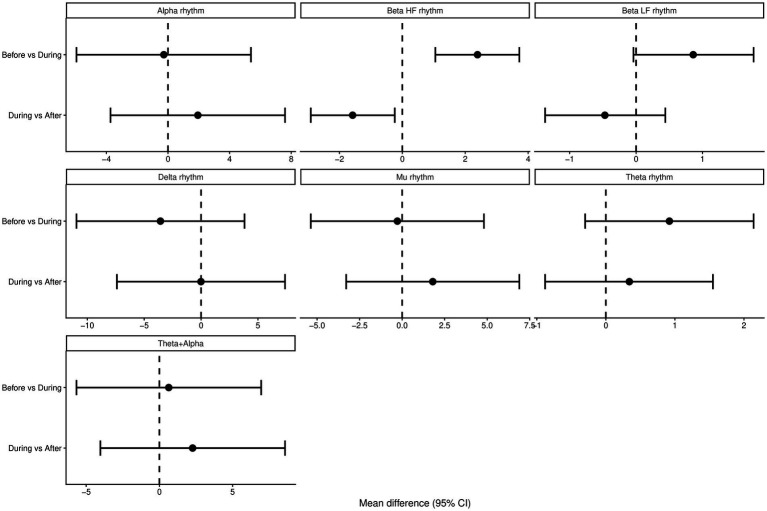
Mean differences between before, during, and after time points across neural rhythm bands (95% CI).

Among the secondary outcomes, high-frequency beta activity (beta HF, 20–30 Hz) was the only frequency band that remained statistically significant after FDR correction. Linear mixed-effects models showed a significant reduction in beta HF power during the sound pill intervention compared with the pre-intervention baseline (Before – During: mean difference = 2.39; 95% CI 1.05 to 3.72; *p* = 0.001; dz = +0.77). Beta HF power during the intervention was also significantly lower than in the post-intervention phase (During – After: mean difference = −1.58; 95% CI −2.91 to −0.24; *p* = 0.023; dz = −0.51). However, the before versus after comparison was not statistically significant (mean difference = 0.81; 95% CI −0.52 to 2.13; *p* = 0.218; dz = +0.26), indicating a transient reduction associated with the intervention period rather than a sustained post-intervention effect (Figure 1; Supplementary Table S1).

**Figure 2:**
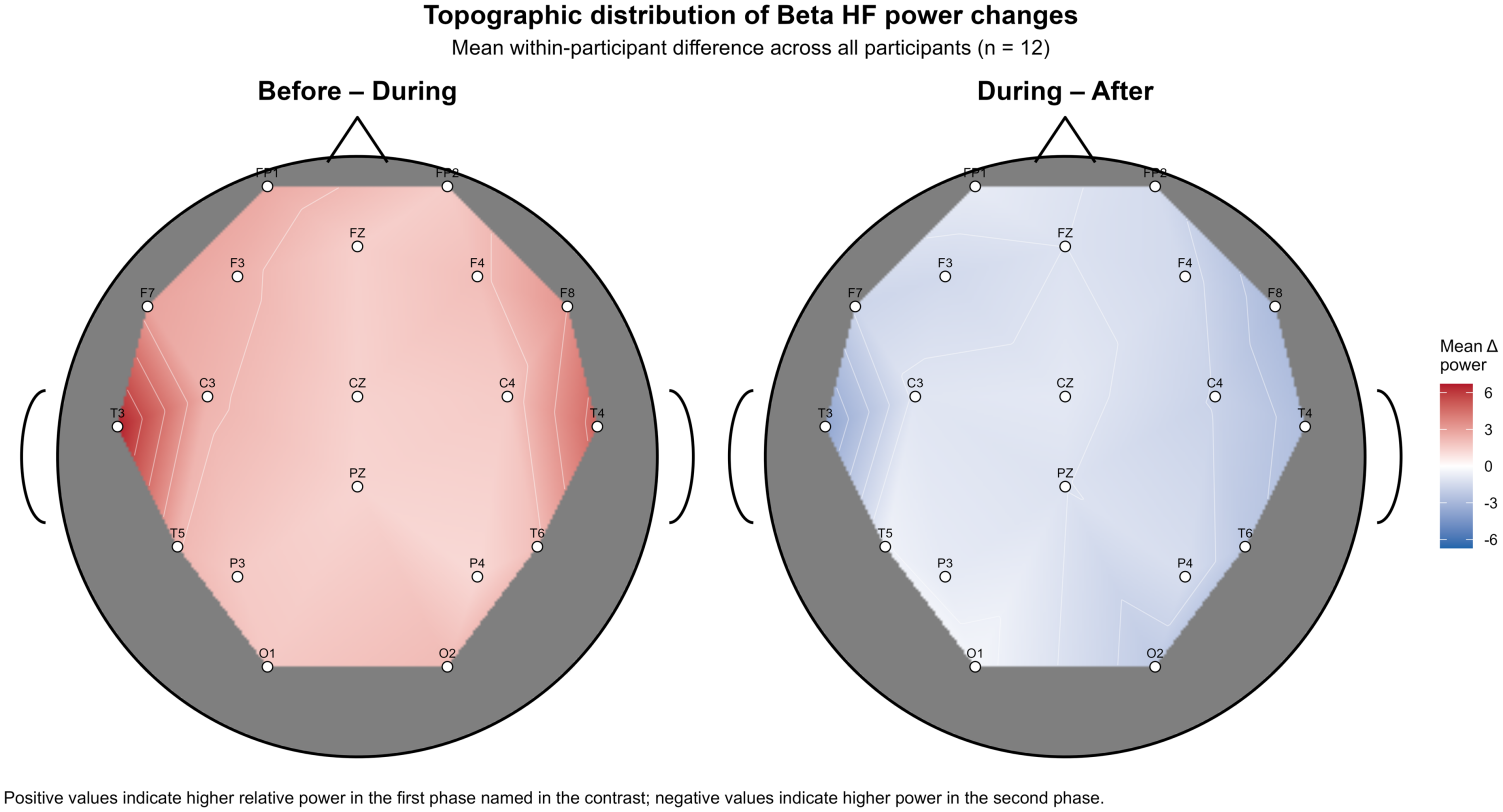
Topographic distribution of high-frequency beta (Beta HF) power changes. Mean within-participant differences are shown for the contrasts Before – During and During – After (*n* = 12). Warm colors indicate higher relative power in the first phase of the contrast; cool colors indicate higher power in the second phase.

To further illustrate the spatial distribution of this effect, topographic maps of Beta HF power changes were generated. These maps showed a relatively widespread reduction in Beta HF activity during the intervention compared with the pre-intervention baseline, followed by a return toward baseline levels in the post-intervention phase (Figure 2).

**Figure 3:**
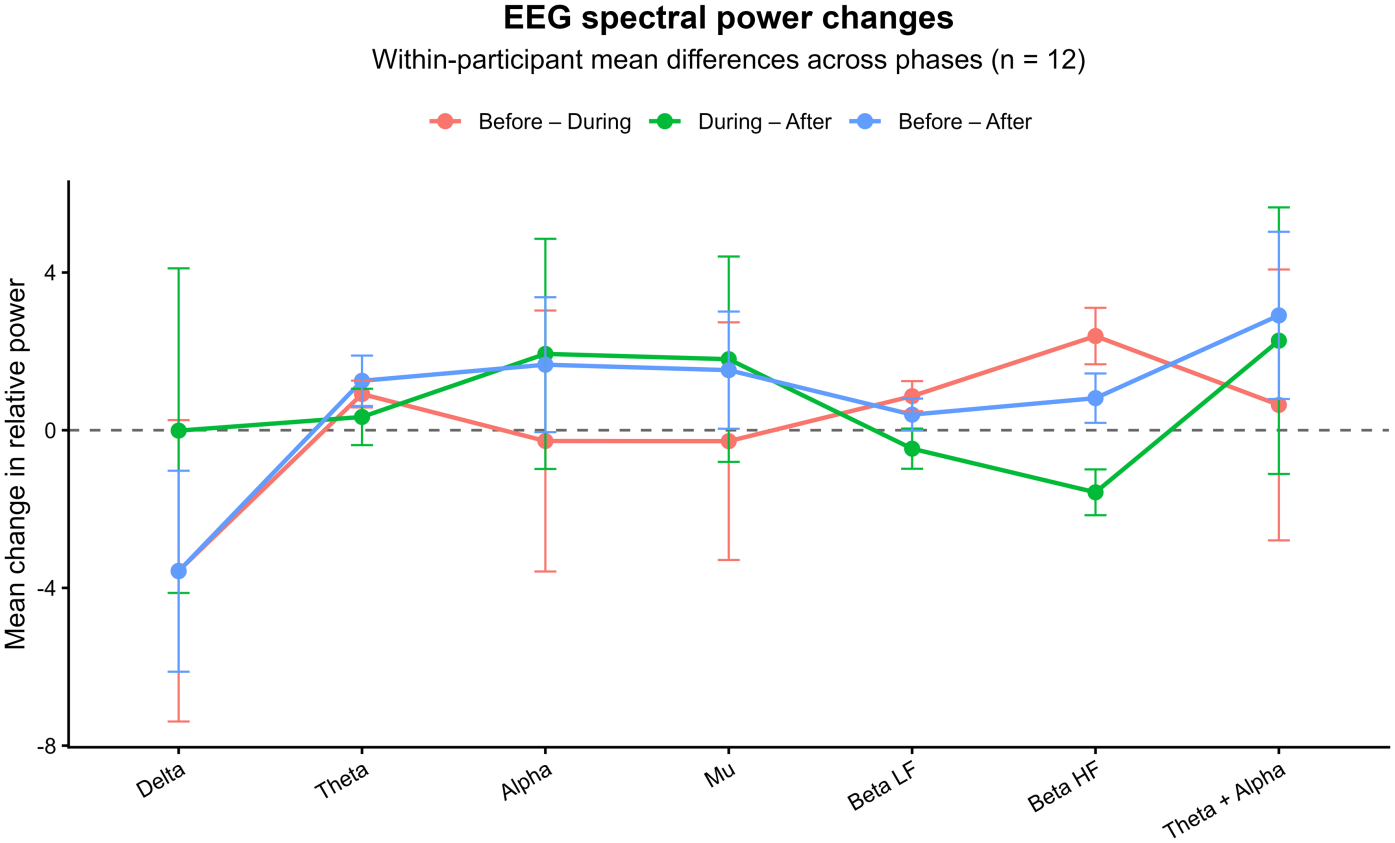
Within-participant mean differences in relative EEG power across frequency bands between experimental phases (*n* = 12). Error bars represent 95% confidence intervals. Positive values indicate higher relative power in the first phase named in the contrast; negative values indicate higher power in the second phase.

To provide an integrated overview of the changes across all frequency bands, within-participant mean differences in relative power were also examined (Figure 3). This visualization confirmed that the most consistent directional change occurred in the Beta HF band.

For descriptive purposes, the mean relative power across frequency bands in each experimental phase is shown in Supplementary Figure S1. Exploratory topographic maps showing power changes in Delta, Theta, Alpha, Mu, Beta LF, and Theta + Alpha bands are provided in Supplementary Figures S2–S7, respectively.

To evaluate the robustness of the aggregated approach, we conducted additional disaggregated analyses comparing the original five conditions. The pattern of results for high-frequency beta activity remained consistent, with a significant reduction observed during the intervention relative to the eyes-closed baseline. For the alpha band, the eyes-closed baseline versus during contrast also yielded a non-significant result, consistent with the primary analysis (Supplementary Table S2). Given the exploratory nature of the study and the small sample size, we retained the aggregated Before/During/After model as the primary analysis. The disaggregated contrasts are reported in the Supplementary material to ensure transparency.

### 3.5 Subgroup analysis

Subgroup analyses were conducted to explore potential modality-specific patterns across the five artistic interventions (visual arts, dance, literature, music, and theater). Given the pilot nature of the study and the very small sample sizes within individual subgroups, these analyses should be interpreted as descriptive and hypothesis-generating rather than confirmatory. Emphasis was placed on the descriptive characterization and directional consistency of observed changes across conditions rather than on formal statistical inference.

#### 3.5.1 Visual arts (*n* = 2)

**Figure 4:**
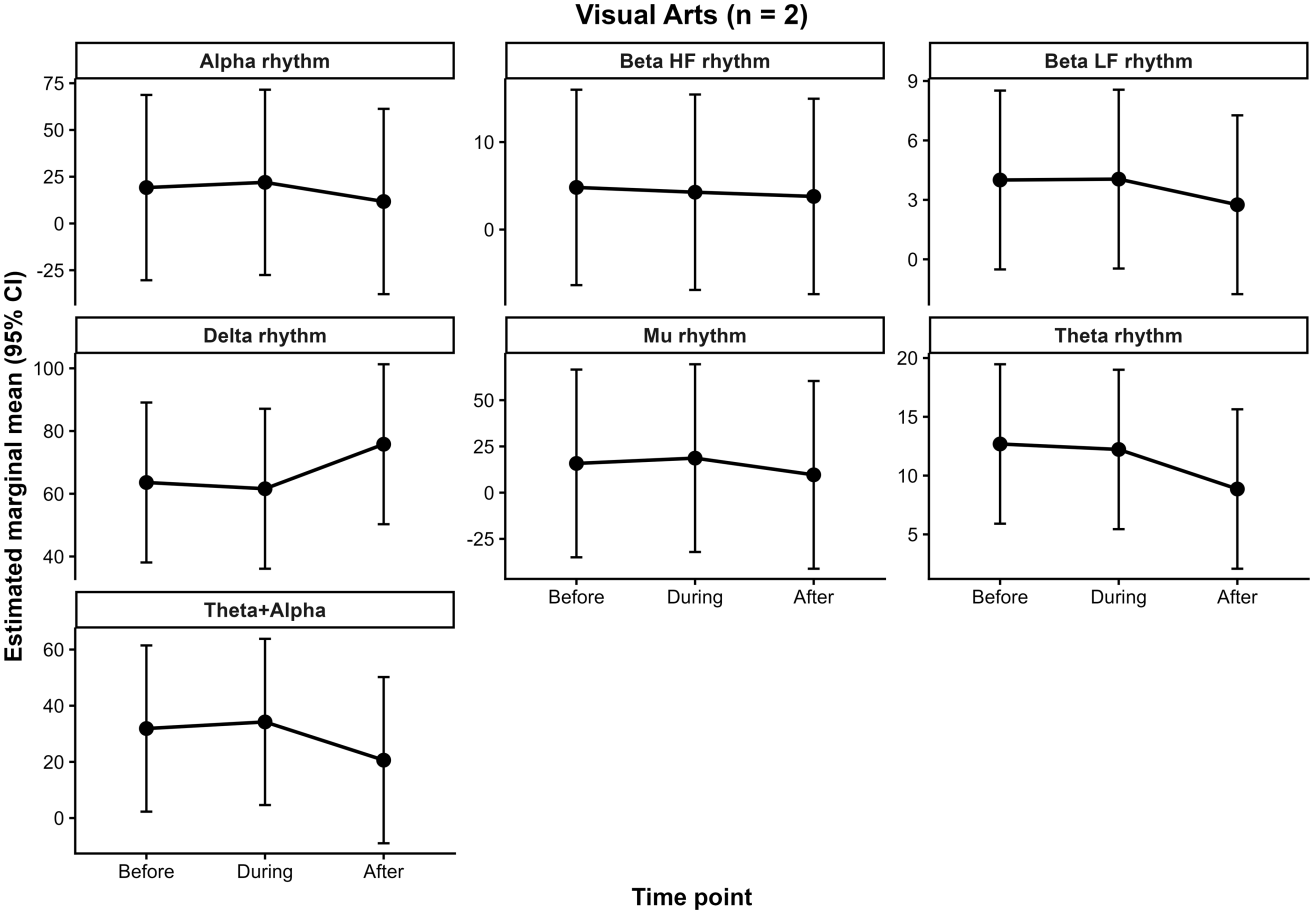
Estimated marginal means of relative power (±95% confidence intervals) in each neural rhythm band across the before, during, and after experimental conditions in the visual arts subgroup (*n* = 2).

In the visual arts subgroup, most oscillatory measures showed a descriptive pattern of lower values in the post-intervention phase compared with baseline, while delta activity tended to increase after the intervention. Given the extremely small sample size (*n* = 2), these observations are highly preliminary and should be interpreted as hypothesis-generating only (Figure 4).

#### 3.5.2 Dance (*n* = 3)

**Figure 5:**
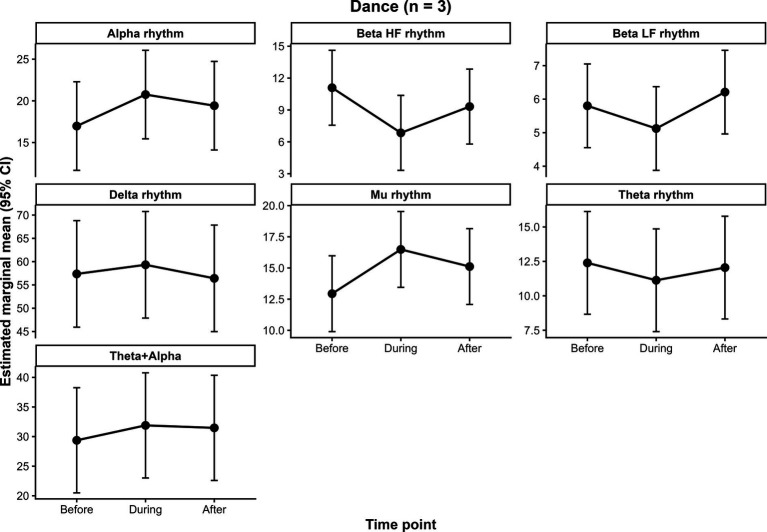
Estimated marginal means of relative power (±95% confidence intervals) in each neural rhythm band across the before, during, and after experimental conditions in the dance subgroup (*n* = 3).

In the dance subgroup, beta HF activity showed descriptively lower values during the intervention compared with baseline, with partial recovery in the post-intervention period. Other measures (alpha, mu, theta, and theta + alpha) exhibited modest and inconsistent fluctuations across conditions. Given the very small sample size, these findings should be viewed as exploratory observations only and not as evidence of reliable modality-specific effects (Figure 5). The high prevalence of anxiety in this subgroup (100%) should be considered when interpreting these patterns.

#### 3.5.3 Literature (*n* = 1)—single-case descriptive data only

The literature modality was represented by a single participant (*n* = 1). Given the absence of replication within this subgroup, no subgroup analysis was performed and no inferential interpretation is possible. Consequently, this modality is not discussed further, and no conclusions regarding literature-specific neurophysiological responses can be drawn from the present study.

#### 3.5.4 Music (*n* = 4)

**Figure 6:**
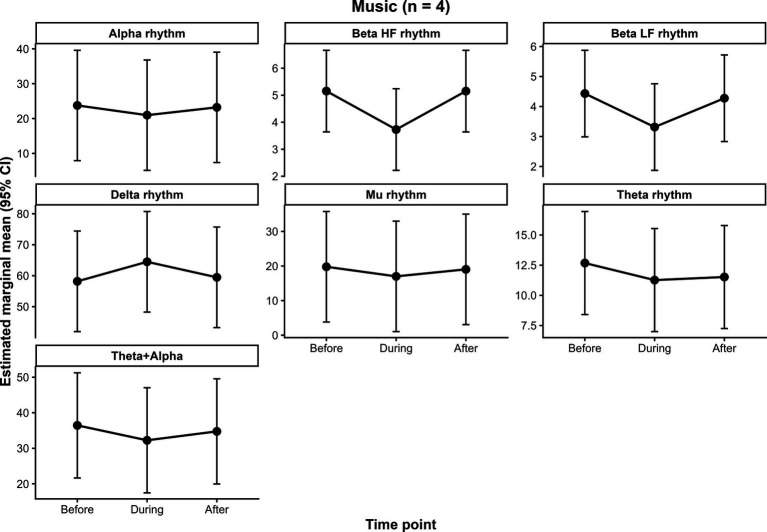
Estimated marginal means of relative power (±95% confidence intervals) in each neural rhythm band across the before, during, and after experimental conditions in the music subgroup (*n* = 4).

In the music subgroup, oscillatory activity remained relatively stable across experimental conditions, with only small descriptive changes observed. Beta HF and beta LF tended to be lower during the intervention, followed by a return toward baseline levels afterward. Given the small sample size and the exploratory nature of the analysis, these observations should be interpreted as hypothesis-generating (Figure 6).

#### 3.5.5 Theater (*n* = 2)

**Figure 7:**
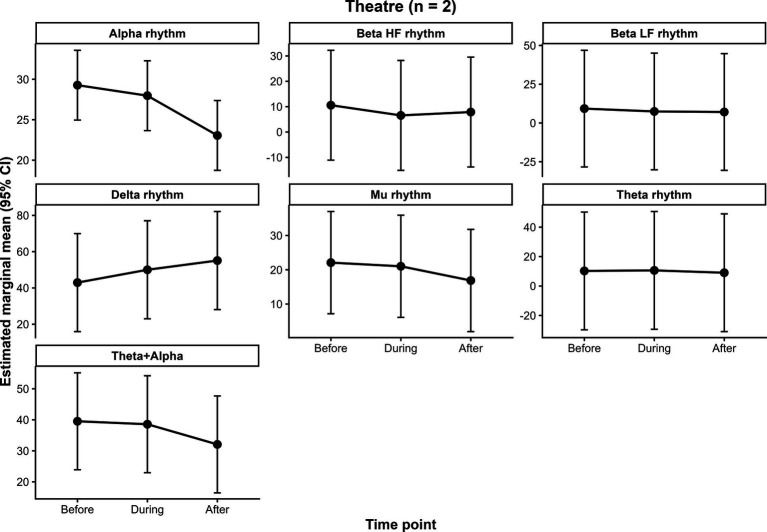
Estimated marginal means of relative power (±95% confidence intervals) in each neural rhythm band across the before, during, and after experimental conditions in the theater subgroup (*n* = 2).

In the theater subgroup, several measures (including alpha, mu, beta HF, beta LF, and theta + alpha) showed descriptively lower values in the post-intervention phase compared with baseline, while delta activity tended to increase progressively across conditions. Given the extremely small sample size (*n* = 2), these patterns are preliminary and should be interpreted with caution (Figure 7).

## 4 Discussion

The findings of this study should be interpreted in light of its exploratory design and relatively small sample size, characteristics typical of pilot investigations aimed at evaluating novel experimental paradigms (Hertzog, 2008; Leon et al., 2011). Although this limits the generalizability of the results, it allows the identification of preliminary neurophysiological patterns and provides a basis for generating more precise hypotheses to be tested in future studies with larger samples and confirmatory designs.

At the group level, high-frequency beta activity (beta HF, 20–30 Hz) was the only secondary outcome to demonstrate statistically significant modulation. Linear mixed-effects models revealed a moderate reduction in beta HF power during the sound pill intervention compared with the pre-intervention baseline, followed by a return toward baseline levels in the post-intervention phase. Supplementary disaggregated analyses indicated that the magnitude of this effect varied according to recording condition (eyes open versus eyes closed), suggesting that state-dependent EEG differences may have partially contributed to the observed beta HF pattern. This finding is best interpreted as a transient stimulus-related reduction, as post-intervention beta HF values did not differ significantly from pre-intervention baseline levels (before vs. after: *p* = 0.218; dz = +0.26), indicating the absence of a sustained post-intervention effect.

Importantly, in the absence of a control condition, such as neutral spoken-word audio, an equivalent eyes-closed rest period, or a non-contemplative soundscape, the observed beta HF modulation cannot be specifically attributed to the artistic–contemplative content of the sound pills. Alternative explanations include sustained auditory attention to an engaging stimulus, cortical beta responses to the prosodic and rhythmic features of narrated speech (Giraud and Poeppel, 2012), or nonspecific time-on-task effects. Accordingly, the present findings should be interpreted as providing preliminary evidence of biological plausibility and protocol feasibility rather than evidence of a specific contemplative–aesthetic neural signature.

In addition, high-frequency beta activity is particularly susceptible to EMG contamination. Because independent component analysis (ICA) was not applied, residual muscular artifacts cannot be ruled out as a partial contributor to the observed beta HF reduction.

Although no statistically significant global effects were observed in the theta and alpha bands, exploratory topographic patterns suggested modality-related differentiations that may reflect distinct subjective dimensions of the experience. Directional increases in mid-frontal theta at Fz and Cz and posterior alpha at Pz, O1, and O2 were observed, together with reduced frontal beta activity, a pattern that may be compatible with reflective processing and internally oriented attention, although such interpretations remain tentative and require confirmation in controlled studies (Klimesch, 1999; Cahn and Polich, 2006; Jensen and Mazaheri, 2010).

Distinct oscillatory patterns were observed across artistic modalities; however, these findings should be interpreted as descriptive and hypothesis-generating given the very small subgroup sample sizes. Visual arts and theater demonstrated post-intervention reductions across several oscillatory measures, particularly alpha, mu, and combined theta + alpha activity, whereas delta activity tended to increase following the intervention. The dance modality showed modest temporal fluctuations, including lower beta HF values during the intervention period. The music modality exhibited relatively stable oscillatory profiles across conditions, with only minor changes over time. Given the exploratory nature of these analyses, it remains unclear whether the observed patterns reflect modality-specific neurophysiological responses or random variation associated with small sample sizes. Future studies with adequately powered samples are required to determine whether distinct artistic modalities produce reproducible oscillatory signatures.

The modality-specific oscillatory patterns should be interpreted with particular caution given the uneven clinical composition of subgroups. Dance and visual arts subgroups both showed 100% prevalence of anxiety diagnoses, and the borderline between-group difference in anxiety prevalence (*p* = 0.061) suggests that differential clinical profiles may have contributed to the observed differences in neural responses. It cannot be excluded that anxiety-related baseline differences in arousal or attentional processing, rather than modality-specific features of the artistic stimulus, account for part of the observed subgroup variation. Future controlled studies should ensure matched clinical profiles across modality conditions or include anxiety status as a covariate.

The absence of any subjective experiential measure constitutes a critical limitation of the present study. The construct of aesthetic states of consciousness is inherently phenomenological, and its neurophysiological correlates can only be meaningfully interpreted in conjunction with experiential reports. Without measures of absorption (e.g., Tellegen Absorption Scale), aesthetic response (e.g., Geneva Appraisal Questionnaire for Music), affect (e.g., PANAS), or perceived presence, the oscillatory changes we report cannot be linked to any specific subjective quality of the aesthetic experience. Future studies must include validated subjective instruments as co-primary outcomes alongside EEG measures.

An important consideration for the potential real-world application of contemplative auditory interventions concerns their effects on cognitive and attentional states relevant to safety. High-frequency beta oscillations have been associated with sustained attention, cognitive vigilance, motor readiness, and externally oriented processing. In contrast, contemplative and internally directed states are often characterized by reduced external sensory engagement and increased internally focused attention. Although the present study was conducted in a controlled laboratory setting with seated participants, interventions that modulate beta activity and promote shifts toward internally oriented processing could theoretically reduce vigilance in contexts that require continuous external monitoring, such as driving, operating machinery, or performing safety-sensitive tasks. Therefore, the sound pills evaluated in this study should not be considered appropriate for use in such situations without further safety assessment. Future translational research should specifically evaluate the effects of these interventions on attentional performance and safety-relevant outcomes before broader implementation is considered.

This study presents several strengths, including the use of a standardized auditory stimulus composed of original scripts combined with structured soundtrack and sound design, the implementation of an EEG protocol organized into multiple temporal phases, and the relative topographic consistency of the observed electrophysiological effects.

### 4.1 Limitations and future directions

Nonetheless, several limitations should be acknowledged. First, the small sample size of 12 participants limited statistical power and reduced the external validity of the findings. Second, the absence of a control condition represents the principal inferential limitation of the study, as the observed effects cannot be specifically attributed to the artistic–contemplative content of the intervention rather than to general auditory engagement or nonspecific session effects.

The high prevalence of psychotropic medication use (67%) constitutes an additional potential confound. Benzodiazepines, selective serotonin reuptake inhibitors (SSRIs), and other psychotropic agents are known to alter EEG spectral power, particularly by influencing beta and alpha oscillatory activity (Blume, 2006). Consequently, part of the observed beta HF modulation may reflect pharmacological influences rather than exclusively contemplative–aesthetic processes. Future studies should either exclude participants using psychotropic medications or perform sensitivity analyses stratified by medication status.

An additional limitation relates to the heterogeneity of the artistic interventions and the imbalance in subgroup sizes across modalities. The distribution of participants across categories such as visual arts, music, dance, literature, and theater was uneven, limiting the ability to perform reliable comparisons between modalities. As a result, the modality-specific patterns observed in the present study should be interpreted as descriptive and hypothesis-generating rather than as robust comparative findings. Future studies with larger and more balanced samples are needed to systematically evaluate potential differences across artistic modalities.

The broad age range of the sample (18–66 years) represents another source of potential heterogeneity. Age-related changes in EEG spectral characteristics are well documented (Dustman et al., 1993; Klimesch, 1999) and may have contributed to interindividual variability, particularly within the theater subgroup (mean age = 45 years, SD = 29.7). Because of the limited sample size, age was not included as a covariate in the inferential models, and its potential influence on the observed EEG patterns cannot be excluded. Future studies should employ larger samples, narrower age ranges, or statistical adjustment for age.

The absence of subjective measures is also a major limitation given the phenomenological nature of the construct under investigation. Finally, the retrospective registration of the clinical trial (ReBEC, June 2025), more than 4 years after ethics approval, should be acknowledged as a limitation related to study registration rigor.

Although the present study was limited to the analysis of EEG power-spectral changes, future investigations would benefit from the incorporation of more advanced computational approaches. Measures of high-order interactions, such as synergy and redundancy derived from partial information decomposition, have recently demonstrated utility in characterizing complex neural interdependencies during non-ordinary states of consciousness, including meditation, hypnosis, and auto-induced cognitive trance (Kumar et al., 2024). Similarly, whole-brain analyses of effective connectivity and information broadcasting have revealed alterations in posterior integration and thalamo-frontotemporal communication in disorders of consciousness (Panda et al., 2023). Furthermore, multimodal approaches combining EEG with fMRI have proven valuable in linking microstate dynamics with default mode network activity during meditation (Panda et al., 2016). The application of these methods in future studies could provide deeper insight into whether the observed reduction in high-frequency beta activity reflects broader changes in directed information flow, network topology, or large-scale brain integration during artistic–contemplative interventions.

Despite these constraints, the findings provide preliminary indications of feasibility and potential neurophysiological effects, which should be further investigated in controlled studies. Future investigations should also incorporate appropriate control conditions and, when raw EEG data are available, apply advanced analytical approaches such as ICA for artifact separation, connectivity metrics, microstate segmentation, and time–frequency decomposition to more comprehensively characterize the underlying neural mechanisms (Cohen, 2014; Michel and Koenig, 2018).

## 5 Conclusion

This exploratory pilot study investigated the neurophysiological effects of mindfulness-based artistic–contemplative “sound pills” in adults using EEG. At the group level, high-frequency beta activity (beta HF) was the only oscillatory band to demonstrate statistically significant modulation, showing a moderate reduction during the intervention compared with baseline. Post-intervention beta HF values did not differ significantly from pre-intervention levels, indicating a transient stimulus-related reduction rather than a sustained contemplative effect. Without a control condition, this finding cannot be specifically attributed to the artistic–contemplative content of the sound pills. No statistically significant global changes were observed in the alpha, theta, delta, mu, or combined theta + alpha bands after FDR correction.

Exploratory subgroup analyses suggested descriptive, modality-specific oscillatory patterns; however, these findings should be interpreted as hypothesis-generating only due to the very small sample sizes per modality and heterogeneous participant characteristics. Visual arts and theater showed descriptive patterns of post-intervention reductions in alpha, mu, and combined theta + alpha activity, alongside increases in delta power. Dance and music exhibited more modest and variable changes. Given the extremely small subgroup sizes and the absence of a control condition, it is not possible to determine whether these patterns reflect true modality-specific responses or random variation. Future studies with larger, clinically homogeneous samples, appropriate control conditions, and validated subjective measures are required before any specific claims about aesthetic states of consciousness can be supported empirically.

## Data Availability

The original contributions presented in the study are included in the article/Supplementary material, further inquiries can be directed to the corresponding author.
