# Supplementary material for: EEG signatures of mindfulness-based artistic–contemplative sound interventions: a pilot study on aesthetic states of consciousness
Source: Front Psychol. 2026 Jul 28;17:1846392. doi: 10.3389/fpsyg.2026.1846392 (PMC13459761; doi:10.3389/fpsyg.2026.1846392)
Supplement: Supplementary file 2 [file Supplementary_file_1.docx]

**Supplementary Material**

**Full Scripts of the Artistic–Contemplative Sound Pills**

The complete scripts used in the mindfulness-based artistic–contemplative sound pill interventions are provided below to ensure transparency and reproducibility of the experimental stimuli.

Each script corresponds to one artistic modality used in the experimental sessions.

**Supplementary Text S1 — Visual Arts**

**The Impressionist Gaze**

This visual arts sound pill explores the concept of the beginner’s mind by inviting participants to engage with perception through an Impressionist perspective inspired by artists such as Monet, Renoir, Manet, and Degas.

Participants are invited to choose a comfortable position—standing, sitting, or lying down—and to close their eyes. The exercise begins with attention directed toward breathing, allowing the flow of inhalation and exhalation to guide the emergence of mental images and sensory impressions.

The intervention encourages participants to imagine themselves walking through the gardens of Giverny, famously painted by Claude Monet. Rather than focusing on rigid outlines or fixed forms, participants are invited to attend to the overall impression of the landscape—its colors, light, movement, and atmosphere.

Drawing on principles similar to Impressionist painting, the exercise emphasizes moment-to-moment perception. Participants are guided to notice subtle changes in light, color, and sensation, much like an artist observing how clouds passing in front of the sun alter the appearance of a scene.

Throughout the experience, participants are encouraged to engage with their sensory channels—vision, hearing, smell, touch, taste, and intuitive perception—while maintaining an open and receptive attitude characteristic of mindfulness practice. This beginner’s gaze invites individuals to experience familiar phenomena as if encountering them for the first time.

As the imagined walk through the gardens continues, participants are invited to observe details such as the height of trees, reflections of sunlight on water, the scent of grass, or the colors of flowers. Each perception is acknowledged and allowed to unfold without judgment, reinforcing attentional awareness and experiential presence.

The intervention concludes by encouraging participants to reflect metaphorically on how they would “paint” their own landscapes of experience. By remaining open to perception and allowing themselves to be impressed by the present moment, participants are invited to cultivate an expanded awareness of their relationship with the surrounding world.

**Supplementary Text S2 — Dance**

**Dancing the Organs**

This dance sound pill invites participants to engage in an internal journey through the body by following the flow of oxygen entering the bloodstream and moving through different organs. The exercise encourages awareness of internal bodily processes while exploring the relationship between the internal and external dimensions of the body.

Participants are invited to choose a comfortable space and remain standing in a relaxed posture with their eyes closed. The experience begins by directing attention to the breath and observing the air entering and leaving the nostrils. As breathing continues, attention is gradually guided through the internal pathways of respiration.

Participants are invited to imagine the path of inhaled air as it moves through the nasal cavity, the larynx, and the trachea, eventually reaching the lungs. The exercise emphasizes awareness of the lungs expanding with each breath. Oxygen is described as entering the bloodstream through the bronchial branches and pulmonary alveoli, small structures that connect the respiratory system to blood circulation.

As breathing continues, participants are encouraged to notice the exchange of gases occurring in the lungs: oxygen entering the bloodstream and carbon dioxide returning to the environment. This process is presented as a continuous cycle linking the body with the surrounding world.

Attention is then directed toward the relationship between the lungs and the heart within the thoracic cavity. Participants are invited to gently place their hands on the chest to perceive the movement of breathing and, if possible, the rhythm of the heartbeat.

The journey then shifts toward the digestive system. Participants are invited to notice the mouth and the movement of the tongue, becoming aware of saliva and the act of swallowing. Attention follows the imagined path of digestion through the esophagus toward the stomach, located on the left side below the rib cage.

The stomach is described as participating in mechanical digestion and initial chemical processing of food. From there, the journey continues through the small intestine, including the jejunum and ileum, where refined chemical digestion and nutrient absorption occur.

Participants are invited to imagine the digestive organs functioning together as part of a coordinated internal process, described metaphorically as a dance that continuously unfolds within the body.

The exercise then introduces additional organs involved in digestion and metabolism, including the liver and gallbladder, which contribute to fat metabolism, as well as the pancreas, which participates in the regulation of sugars through digestive enzymes.

Attention continues through the large intestine, which completes digestion and prepares waste for elimination. The different sections of the colon—ascending, transverse, and descending—are briefly described, followed by the rectum, which completes the digestive cycle.

The kidneys are then introduced as organs responsible for filtering the blood and removing impurities through urine production. Participants are invited to imagine the kidneys connected through the ureters to the bladder, where urine is temporarily stored before elimination.

The exercise also acknowledges reproductive organs as part of the body’s internal system, noting the presence of the uterus and ovaries in women and the prostate in men.

In the final phase of the exercise, participants are invited to gradually initiate subtle movements inspired by internal sensations. These movements may begin as small, delicate internal impulses and progressively extend to the limbs and spine.

Participants are encouraged to allow the body to move freely without concern for form or correctness, maintaining awareness of breathing and internal volume during the movement.

The experience concludes by inviting participants to pause and observe their bodily sensations, reflecting on the relationship between the internal and external dimensions of the body and noticing how the body feels after the movement experience.

**Supplementary Text S3 — Literature**

**The Odyssey and the Song of the Sirens**

This literature sound pill invites participants to reflect on the symbolic episode of the Song of the Sirens from Homer’s Odyssey. The image of Odysseus tied to the central mast of the ship, with his ears open while his companions remain protected, serves as a powerful metaphor for curiosity, knowledge, and the search for self-understanding during the human journey.

Participants are invited to choose a comfortable position—standing, sitting, or lying down—and to close their eyes. The exercise begins with a guided visualization of an ocean journey, representing the metaphorical voyage of human life and the search for one’s personal destination, symbolized by Ithaca.

Attention is directed to breathing while participants imagine themselves navigating across a vast sea. The metaphor emphasizes that, although external conditions such as winds and tides may guide the journey, individuals remain responsible for determining their direction and destination.

The narrative introduces the Odyssey, written by Homer approximately three thousand years ago, which describes the long return journey of Odysseus after the Trojan War. In this literary tradition, Odysseus becomes a symbolic figure representing the human search for identity, knowledge, and fulfillment. The journey toward Ithaca is presented as a metaphor for the human process of self-realization.

Participants are invited to consider the famous episode of the Sirens, mythical beings whose enchanting song lured sailors toward destruction. In mythological symbolism, the Sirens represent powerful desires, temptations, and instinctive impulses that can lead individuals away from their intended paths.

Before Odysseus encounters the Sirens, the sorceress Circe warns him of the danger they represent. She advises him to protect his companions by sealing their ears with wax so that they cannot hear the Sirens’ song. However, she suggests a different strategy for Odysseus himself: he should be tied to the mast of the ship so that he can safely listen to the song without being able to act upon its seductive call.

This image becomes a central metaphor within the exercise. Odysseus tied to the mast—with his ears open—symbolizes a balanced attitude toward curiosity, knowledge, and experience. Rather than avoiding the call of the unknown, the story suggests that wisdom may lie in engaging with it consciously and with appropriate safeguards.

Participants are invited to reflect on the tension between caution and curiosity. Blocking one’s ears entirely may seem like the safest choice, but it also prevents exposure to knowledge and experience. Conversely, allowing oneself to hear the Sirens’ song without guidance may lead to loss of direction.

Within this symbolic framework, the narrative suggests that the human journey requires both openness and discernment. True prudence is presented as a subtle balance between exploration and self-regulation.

The Odyssey therefore becomes a broader metaphor for human life: each individual undertakes their own journey, facing challenges, temptations, and discoveries while seeking meaning and fulfillment.

The exercise concludes by inviting participants to reflect on the path they are navigating in their own lives. Just as Odysseus journeys toward Ithaca, individuals are encouraged to recognize that the process of becoming fully human involves not only reaching a destination but also appreciating the beauty and significance of the path itself.

**Supplementary Text S4 — Music**

**Time**

This music sound pill invites participants to reflect on the concept of time through the lens of musical experience. Music is understood as the art of organizing sounds and silences within time, and rhythm emerges as the most fundamental element that structures this temporal experience.

Participants are invited to choose a comfortable position—standing, sitting, or lying down—and to close their eyes. The exercise begins with attention directed toward breathing, encouraging participants to notice the rhythm of inhalation and exhalation.

The intervention introduces the idea that music exists through time as a dynamic and ephemeral art form. Unlike static arts such as painting or sculpture, musical works unfold through movement and duration, becoming present only while they are experienced. In this sense, music belongs to the family of dynamic arts, alongside theater and dance.

Within music, rhythm represents the fundamental structure through which time becomes perceptible. Rhythm organizes the sequence of sounds and silences, giving shape to musical experience. Participants are invited to consider how rhythm operates not only in music but also within the human body.

The concept of pulse is introduced as the underlying structure of rhythm. In musical terms, the pulse organizes notes, melodies, and harmonies. In biological terms, the pulse can be associated with the beating of the human heart. Participants are encouraged to reflect on their own bodily rhythm and to notice how their internal pulse may relate to their current state of experience.

The exercise explores how pulse structures musical expression by determining the spacing between sounds as well as the silences that occur between them. These intervals contribute to the perception of continuity and movement within music.

Participants are introduced to the concept of tempo, which describes the speed of the musical pulse. Tempo may be measured objectively through devices such as the metronome, which provides a precise number of beats per minute. However, musical tempo may also be expressed through qualitative indications such as allegro, andante, presto, or adagio, which historically preceded the invention of the metronome and convey expressive character as well as speed.

This distinction highlights that musical time is not only quantitative but also experiential and interpretative. For example, moving slowly is not necessarily the same as moving calmly, and moving quickly is not necessarily the same as being rushed.

Participants are therefore invited to reflect on the “tempo” of their own lives. They may consider whether their current rhythm feels accelerated, slowed, or balanced, and how internal states may influence the perceived pace of experience.

The exercise also introduces the idea that tempo and pulse are not always rigid. In many musical traditions, particularly after the innovations of composers such as Beethoven and later Impressionist composers, the pulse may become flexible and expressive rather than strictly regular. In these contexts, musical rhythm may resemble natural processes such as breathing, ocean waves, or the movement of trees in the wind.

Through this metaphor, the exercise invites participants to recognize that temporal experience in human life may also fluctuate. Just as musical rhythm can expand or contract, the rhythms of daily life may change according to circumstances, emotions, and contexts.

The intervention concludes by encouraging participants to reflect on their current rhythm of life and to consider whether adjustments—such as slowing down, maintaining balance, or allowing natural fluctuations—may support a more attentive and conscious relationship with time.

**Supplementary Text S5 — Theatre**

**Hamlet: On the Dilemma of “To Be or Not to Be”**

This theatre sound pill invites participants to explore the functioning of their own thoughts through reflection on the existential dilemma presented in William Shakespeare’s Hamlet. The famous question “To be or not to be” serves as a starting point for examining inner dialogue, uncertainty, and the human tendency to reflect on existence, choice, and control.

Participants are invited to choose a comfortable position—standing, sitting, or lying down—and to close their eyes. The exercise begins with attention directed toward breathing and observation of the present mental state.

Participants are encouraged to observe their thoughts as they arise. Thoughts may appear as words, images, or even as moments of apparent silence in which one wonders whether thoughts are present at all. This process invites reflection on the spontaneous and often automatic nature of thinking.

During the exercise, participants are guided to notice the internal voice that comments on experiences, interprets events, and responds to external stimuli. The intervention highlights how thoughts can emerge rapidly and continuously, often without deliberate intention. Participants are therefore invited to question whether these thoughts are actively controlled or whether they arise as part of the mind’s natural activity.

The narrative then introduces Hamlet, one of the most widely performed works in the history of theatre, written by William Shakespeare over four centuries ago. The play tells the story of Prince Hamlet, who is confronted with the task of avenging his father’s death after discovering that his uncle Claudius has taken both the throne and the queen.

Within the play, Hamlet’s famous soliloquy—“To be or not to be”—expresses a profound reflection on the meaning of existence. In this passage, Hamlet contemplates whether it is more honorable to endure the difficulties of life or to resist them by ending one’s suffering.

In the context of this exercise, the dilemma serves as a metaphor for the human tendency toward reflection, hesitation, and internal deliberation. Participants are invited to consider how excessive analysis may sometimes lead individuals to become trapped in cycles of thought that delay action or decision-making.

At the same time, the narrative highlights that reflection can also create awareness. Observing the mind allows individuals to recognize that many internal experiences—such as doubt, fear, or desire—arise naturally and fluctuate over time.

The exercise therefore invites participants to observe their thoughts without attempting to suppress or control them. Instead, they are encouraged to notice how thoughts emerge, change, and eventually pass, much like other transient mental experiences.

In its final reflection, the intervention emphasizes that human life contains inherent oppositions: joy and sadness, health and illness, beginnings and endings. Rather than avoiding difficult experiences, individuals may cultivate awareness by acknowledging these complementary aspects of existence.

The exercise concludes by inviting participants to remain present with their current experience and to recognize that awareness itself—simply noticing thoughts and sensations as they arise—can offer a meaningful way to engage with the complexities of life.

**Supplementary Table S1.** Pairwise comparisons between experimental conditions for each EEG rhythm band.

| **Band** | **Comparison** | **Mean difference** | **SE** | **df** | **t** | **p value** | **95% CI (lower)** | **95% CI (upper)** | **Cohen’s dz** |
| --- | --- | --- | --- | --- | --- | --- | --- | --- | --- |
| Alpha rhythm | Before – During | -0.27 | 2.73 | 22 | -0.10 | 0.921 | -5.94 | 5.39 | -0.02 |
| Alpha rhythm | Before – After | 1.66 | 2.73 | 22 | 0.61 | 0.549 | -4.01 | 7.33 | 0.13 |
| Alpha rhythm | During – After | 1.94 | 2.73 | 22 | 0.71 | 0.486 | -3.73 | 7.60 | 0.15 |
| Beta HF rhythm | Before – During | 2.39 | 0.64 | 22 | 3.71 | 0.001 | 1.05 | 3.72 | 0.77 |
| Beta HF rhythm | Before – After | 0.81 | 0.64 | 22 | 1.26 | 0.220 | -0.52 | 2.15 | 0.26 |
| Beta HF rhythm | During – After | -1.58 | 0.64 | 22 | -2.45 | 0.023 | -2.91 | -0.24 | -0.51 |
| Beta LF rhythm | Before – During | 0.86 | 0.44 | 22 | 1.98 | 0.060 | -0.04 | 1.77 | 0.41 |
| Beta LF rhythm | Before – After | 0.40 | 0.44 | 22 | 0.91 | 0.374 | -0.51 | 1.30 | 0.19 |
| Beta LF rhythm | During – After | -0.47 | 0.44 | 22 | -1.07 | 0.294 | -1.37 | 0.44 | -0.22 |
| Delta rhythm | Before – During | -3.57 | 3.56 | 22 | -1.00 | 0.328 | -10.95 | 3.82 | -0.21 |
| Delta rhythm | Before – After | -3.58 | 3.56 | 22 | -1.00 | 0.326 | -10.96 | 3.81 | -0.21 |
| Delta rhythm | During – After | -0.01 | 3.56 | 22 | -0.00 | 0.998 | -7.40 | 7.38 | -0.00 |
| Mu rhythm | Before – During | -0.28 | 2.45 | 22 | -0.11 | 0.911 | -5.37 | 4.81 | -0.02 |
| Mu rhythm | Before – After | 1.52 | 2.45 | 22 | 0.62 | 0.541 | -3.57 | 6.61 | 0.13 |
| Mu rhythm | During – After | 1.80 | 2.45 | 22 | 0.73 | 0.471 | -3.29 | 6.89 | 0.15 |
| Theta rhythm | Before – During | 0.92 | 0.59 | 22 | 1.56 | 0.133 | -0.30 | 2.14 | 0.33 |
| Theta rhythm | Before – After | 1.25 | 0.59 | 22 | 2.13 | 0.044 | 0.04 | 2.47 | 0.45 |
| Theta rhythm | During – After | 0.34 | 0.59 | 22 | 0.57 | 0.573 | -0.88 | 1.55 | 0.12 |
| Theta + Alpha | Before – During | 0.64 | 3.04 | 22 | 0.21 | 0.835 | -5.66 | 6.95 | 0.04 |
| Theta + Alpha | Before – After | 2.91 | 3.04 | 22 | 0.96 | 0.349 | -3.39 | 9.22 | 0.20 |
| Theta + Alpha | During – After | 2.27 | 3.04 | 22 | 0.75 | 0.463 | -4.03 | 8.58 | 0.16 |

**Notes**

SE = standard error of the mean difference.

df = degrees of freedom.

CI = confidence interval.

Cohen’s dz = standardized effect size for paired comparisons.

Comparisons refer to the three experimental phases of the intervention:

Before (baseline measurement before the sound pill),

During (measurement during the sound pill), and

After (measurement after the sound pill).

All analyses correspond to paired comparisons derived from the linear mixed-effects model described in the Methods section.

FDR correction was applied within each frequency band. Nominal (uncorrected) p-values are presented for transparency; statistical significance was determined based on FDR-corrected results.

**Supplementary Table S2.** Pairwise contrasts from the disaggregated analysis (5 conditions: eyes-open baseline, eyes-closed baseline, during-intervention, eyes-open post-intervention, and eyes-closed post-intervention).

This table presents estimated marginal mean differences between eyes-open (OA) and eyes-closed (OF) recordings at baseline and post-intervention, as well as comparisons involving the during-intervention phase. It was conducted as a robustness check following reviewer feedback regarding the aggregation of eyes-open and eyes-closed recordings in the main analyses.

| **Band** | **Contrast** | **Estimate** | **SE** | **df** | **t** | **p** | **Cohen's dz** |
| --- | --- | --- | --- | --- | --- | --- | --- |
| Alpha rhythm | OA Before - OF Before | -6.803 | 3.352 | 44 | -2.030 | 0.048 | -0.306 |
| Alpha rhythm | OA Before - During | -3.675 | 3.352 | 44 | -1.097 | 0.279 | -0.165 |
| Alpha rhythm | OA Before - OA After | 0.827 | 3.352 | 44 | 0.247 | 0.806 | 0.037 |
| Alpha rhythm | OA Before - OF After | -4.306 | 3.352 | 44 | -1.285 | 0.206 | -0.194 |
| Alpha rhythm | OF Before - During | 3.128 | 3.352 | 44 | 0.933 | 0.356 | 0.141 |
| Alpha rhythm | OF Before - OA After | 7.630 | 3.352 | 44 | 2.276 | 0.028 | 0.343 |
| Alpha rhythm | OF Before - OF After | 2.497 | 3.352 | 44 | 0.745 | 0.460 | 0.112 |
| Alpha rhythm | During - OA After | 4.502 | 3.352 | 44 | 1.343 | 0.186 | 0.203 |
| Alpha rhythm | During - OF After | -0.631 | 3.352 | 44 | -0.188 | 0.852 | -0.028 |
| Alpha rhythm | OA After - OF After | -5.133 | 3.352 | 44 | -1.531 | 0.133 | -0.231 |
| Beta HF rhythm | OA Before - OF Before | 1.767 | 0.793 | 44 | 2.228 | 0.031 | 0.336 |
| Beta HF rhythm | OA Before - During | 3.271 | 0.793 | 44 | 4.126 | < .001 | 0.622 |
| Beta HF rhythm | OA Before - OA After | 0.076 | 0.793 | 44 | 0.096 | 0.924 | 0.015 |
| Beta HF rhythm | OA Before - OF After | 3.317 | 0.793 | 44 | 4.183 | < .001 | 0.631 |
| Beta HF rhythm | OF Before - During | 1.505 | 0.793 | 44 | 1.898 | 0.064 | 0.286 |
| Beta HF rhythm | OF Before - OA After | -1.690 | 0.793 | 44 | -2.132 | 0.039 | -0.321 |
| Beta HF rhythm | OF Before - OF After | 1.550 | 0.793 | 44 | 1.955 | 0.057 | 0.295 |
| Beta HF rhythm | During - OA After | -3.195 | 0.793 | 44 | -4.030 | < .001 | -0.607 |
| Beta HF rhythm | During - OF After | 0.045 | 0.793 | 44 | 0.057 | 0.955 | 0.009 |
| Beta HF rhythm | OA After - OF After | 3.240 | 0.793 | 44 | 4.087 | < .001 | 0.616 |
| Beta LF rhythm | OA Before - OF Before | 1.018 | 0.546 | 44 | 1.865 | 0.069 | 0.281 |
| Beta LF rhythm | OA Before - During | 1.373 | 0.546 | 44 | 2.516 | 0.016 | 0.379 |
| Beta LF rhythm | OA Before - OA After | -0.040 | 0.546 | 44 | -0.073 | 0.942 | -0.011 |
| Beta LF rhythm | OA Before - OF After | 1.849 | 0.546 | 44 | 3.388 | 0.001 | 0.511 |
| Beta LF rhythm | OF Before - During | 0.355 | 0.546 | 44 | 0.650 | 0.519 | 0.098 |
| Beta LF rhythm | OF Before - OA After | -1.058 | 0.546 | 44 | -1.939 | 0.059 | -0.292 |
| Beta LF rhythm | OF Before - OF After | 0.831 | 0.546 | 44 | 1.522 | 0.135 | 0.229 |
| Beta LF rhythm | During - OA After | -1.413 | 0.546 | 44 | -2.589 | 0.013 | -0.390 |
| Beta LF rhythm | During - OF After | 0.476 | 0.546 | 44 | 0.872 | 0.388 | 0.131 |
| Beta LF rhythm | OA After - OF After | 1.889 | 0.546 | 44 | 3.461 | 0.001 | 0.522 |
| Delta rhythm | OA Before - OF Before | 3.365 | 3.991 | 44 | 0.843 | 0.404 | 0.127 |
| Delta rhythm | OA Before - During | -1.883 | 3.991 | 44 | -0.472 | 0.639 | -0.071 |
| Delta rhythm | OA Before - OA After | -1.178 | 3.991 | 44 | -0.295 | 0.769 | -0.044 |
| Delta rhythm | OA Before - OF After | -2.610 | 3.991 | 44 | -0.654 | 0.517 | -0.099 |
| Delta rhythm | OF Before - During | -5.248 | 3.991 | 44 | -1.315 | 0.195 | -0.198 |
| Delta rhythm | OF Before - OA After | -4.543 | 3.991 | 44 | -1.138 | 0.261 | -0.172 |
| Delta rhythm | OF Before - OF After | -5.975 | 3.991 | 44 | -1.497 | 0.142 | -0.226 |
| Delta rhythm | During - OA After | 0.705 | 3.991 | 44 | 0.177 | 0.861 | 0.027 |
| Delta rhythm | During - OF After | -0.727 | 3.991 | 44 | -0.182 | 0.856 | -0.027 |
| Delta rhythm | OA After - OF After | -1.432 | 3.991 | 44 | -0.359 | 0.721 | -0.054 |
| Mu rhythm | OA Before - OF Before | -6.711 | 3.084 | 44 | -2.176 | 0.035 | -0.328 |
| Mu rhythm | OA Before - During | -3.633 | 3.084 | 44 | -1.178 | 0.245 | -0.178 |
| Mu rhythm | OA Before - OA After | 1.013 | 3.084 | 44 | 0.328 | 0.744 | 0.050 |
| Mu rhythm | OA Before - OF After | -4.677 | 3.084 | 44 | -1.516 | 0.137 | -0.229 |
| Mu rhythm | OF Before - During | 3.078 | 3.084 | 44 | 0.998 | 0.324 | 0.150 |
| Mu rhythm | OF Before - OA After | 7.724 | 3.084 | 44 | 2.504 | 0.016 | 0.378 |
| Mu rhythm | OF Before - OF After | 2.034 | 3.084 | 44 | 0.660 | 0.513 | 0.099 |
| Mu rhythm | During - OA After | 4.646 | 3.084 | 44 | 1.506 | 0.139 | 0.227 |
| Mu rhythm | During - OF After | -1.043 | 3.084 | 44 | -0.338 | 0.737 | -0.051 |
| Mu rhythm | OA After - OF After | -5.689 | 3.084 | 44 | -1.845 | 0.072 | -0.278 |
| Theta rhythm | OA Before - OF Before | 0.815 | 0.831 | 44 | 0.981 | 0.332 | 0.148 |
| Theta rhythm | OA Before - During | 1.325 | 0.831 | 44 | 1.595 | 0.118 | 0.240 |
| Theta rhythm | OA Before - OA After | 0.408 | 0.831 | 44 | 0.491 | 0.626 | 0.074 |
| Theta rhythm | OA Before - OF After | 2.914 | 0.831 | 44 | 3.507 | 0.001 | 0.529 |
| Theta rhythm | OF Before - During | 0.510 | 0.831 | 44 | 0.613 | 0.543 | 0.092 |
| Theta rhythm | OF Before - OA After | -0.407 | 0.831 | 44 | -0.490 | 0.627 | -0.074 |
| Theta rhythm | OF Before - OF After | 2.099 | 0.831 | 44 | 2.526 | 0.015 | 0.381 |
| Theta rhythm | During - OA After | -0.917 | 0.831 | 44 | -1.103 | 0.276 | -0.166 |
| Theta rhythm | During - OF After | 1.589 | 0.831 | 44 | 1.912 | 0.062 | 0.288 |
| Theta rhythm | OA After - OF After | 2.506 | 0.831 | 44 | 3.016 | 0.004 | 0.455 |
| Theta+Alpha | OA Before - OF Before | -5.985 | 3.636 | 44 | -1.646 | 0.107 | -0.248 |
| Theta+Alpha | OA Before - During | -2.351 | 3.636 | 44 | -0.647 | 0.521 | -0.097 |
| Theta+Alpha | OA Before - OA After | 1.234 | 3.636 | 44 | 0.339 | 0.736 | 0.051 |
| Theta+Alpha | OA Before - OF After | -1.395 | 3.636 | 44 | -0.384 | 0.703 | -0.058 |
| Theta+Alpha | OF Before - During | 3.633 | 3.636 | 44 | 0.999 | 0.323 | 0.151 |
| Theta+Alpha | OF Before - OA After | 7.219 | 3.636 | 44 | 1.986 | 0.053 | 0.299 |
| Theta+Alpha | OF Before - OF After | 4.590 | 3.636 | 44 | 1.262 | 0.213 | 0.190 |
| Theta+Alpha | During - OA After | 3.586 | 3.636 | 44 | 0.986 | 0.329 | 0.149 |
| Theta+Alpha | During - OF After | 0.957 | 3.636 | 44 | 0.263 | 0.794 | 0.040 |
| Theta+Alpha | OA After - OF After | -2.629 | 3.636 | 44 | -0.723 | 0.473 | -0.109 |
| Note. OA = eyes-open; OF = eyes-closed. Cohen’s dz was approximated as t / √df from the linear mixed-effects model contrast (Satterthwaite degrees of freedom). This provides a standardized effect size for within-subject paired comparisons derived from the model. Note that this is an approximation commonly reported in LMM contrast tables; exact computation via dedicated functions (e.g., emmeans::eff_size()) may be considered in future analyses. Positive values indicate an increase from the first to the second condition, consistent with the sign convention used throughout the manuscript. | | | | | | | |

**Exploratory and descriptive analyses**

**Supplementary Figure S1.** Mean relative EEG power across frequency bands in each experimental phase. Mean relative power (± standard error) in each frequency band during the Before, During, and After phases (n = 12). Before and After phases combine eyes-open and eyes-closed recordings.

**Supplementary Figure S2.** Topographic distribution of Delta power changes. Mean within-participant differences in relative Delta power are shown for the contrasts Before – During and During – After (n = 12). Warm colors indicate higher relative power in the first phase of the contrast; cool colors indicate higher power in the second phase.

**Supplementary Figure S3.** Topographic distribution of Theta power changes. Mean within-participant differences in relative Theta power are shown for the contrasts Before – During and During – After (n = 12). Warm colors indicate higher relative power in the first phase of the contrast; cool colors indicate higher power in the second phase.

**Supplementary Figure S4.** Topographic distribution of Alpha power changes. Mean within-participant differences in relative Alpha power are shown for the contrasts Before – During and During – After (n = 12). Warm colors indicate higher relative power in the first phase of the contrast; cool colors indicate higher power in the second phase.

**Supplementary Figure S5.** Topographic distribution of Mu power changes. Mean within-participant differences in relative Mu power are shown for the contrasts Before – During and During – After (n = 12). Warm colors indicate higher relative power in the first phase of the contrast; cool colors indicate higher power in the second phase.

**Supplementary Figure S6.** Topographic distribution of Beta LF power changes. Mean within-participant differences in relative low-frequency beta (Beta LF) power are shown for the contrasts Before – During and During – After (n = 12). Warm colors indicate higher relative power in the first phase of the contrast; cool colors indicate higher power in the second phase.

**Supplementary Figure S7.** Topographic distribution of Theta + Alpha power changes. Mean within-participant differences in relative combined Theta + Alpha power are shown for the contrasts Before – During and During – After (n = 12). Warm colors indicate higher relative power in the first phase of the contrast; cool colors indicate higher power in the second phase.
